# Differentiation of Goat Meat Freshness Using Gas Chromatography with Ion Mobility Spectrometry

**DOI:** 10.3390/molecules28093874

**Published:** 2023-05-04

**Authors:** Shan He, Bin Zhang, Xuan Dong, Yuqing Wei, Hongtu Li, Bo Tang

**Affiliations:** College of Food and Bioengineering, Bengbu University, Bengbu 233000, China

**Keywords:** goat meat, GC-IMS, storage time, volatile components, PCA

## Abstract

To investigate the flavor changes in goat meat upon storage, the volatile components observed in goat meat after different storage periods were determined using gas chromatography–ion mobility spectrometry (GC–IMS). A total of 38 volatile organic compounds (VOCs) were determined from the goat meat samples, including alcohols, ketones, aldehydes, esters, hydrocarbons, ethers, and amine compounds. 1-Hexanol, 3-Hydroxy-2-butanone, and Ethyl Acetate were the main volatile substances in fresh goat meat, and they rapidly decreased with increasing storage time and can be used as biomarkers for identifying fresh meat. When combined with the contents of total volatile basic–nitrogen (TVB-N) and the total numbers of bacterial colonies observed in physical and chemical experiments, the characteristic volatile components of fresh, sub-fresh, and spoiled meat were determined by principal component analysis (PCA). This method will help with the detection of fraudulent production dates in goat meat sales.

## 1. Introduction

Goat meat is a high-quality meat source that is not only nutritious but also low in fat, cholesterol, and saturated fatty acids when compared to beef and pork [1,2,3]. However, the physiological and biochemical metabolism of goat meat after slaughter quickly changes the quality of fresh meat, and the phenomena of rotting, discoloration, and staling readily occur in the circulation chain of storage, transit, and sale [4,5,6]. Freshness is the fundamental metric used to assess the nutritive value and security of meat products for sellers and consumers [7,8]. Physical and chemical testing or microbial experiments are highly accurate, but the operation processes are cumbersome, the experimental conditions are demanding, and the tests are time-consuming and laborious [9]. Although sensory analysis is simple, the outcomes of this type of evaluation are frequently subjective and cannot provide accurate data for quantitative or qualitative analysis [10].

The rapid identification of the freshness of meat products is an important topic in food research. Nowadays, instrumental analytical technologies, including electronic nose [11,12], spectroscopy [13,14,15], gas chromatography-olfactometry (GC-O) [16,17], and chromatography–mass spectrometry [18,19,20], etc., have been widely used in food quality characterization, such as food flavor detection, adulteration traceability, and other applications. GC-IMS technology combines the high separation capacity of gas chromatography and the fast response of ion mobility spectrometry [21]. When compared with an electronic nose and gas chromatography–mass spectrometry (GC-MS), GC-IMS overcomes the poor qualitative accuracy of GC, difficult separation of cross mixtures, and low sensitivity of complex mixture analysis [22,23]. Headspace-gas chromatography (HS-GC) coupled with ion mobility spectrometry (IMS) has been proposed as an alternative to plate counting to detect and quantify microbial contamination in pomegranate juice [24]. Research toward establishing fingerprint analysis using GC-IMS technology combined with stoichiometry has progressed rapidly [25,26,27,28,29,30,31]. For example, Tian et al. reported the use of GC and GC-IMS technologies to detect peanut oil (PO) adulterated with rapeseed oil (RO) in varying ratios, with good adulteration determination results obtained using GC-IMS in conjunction with principal component analysis (PCA) and component analysis (CA) [32]. Chen et al. employed GC-IMS to rapidly and non-destructively profile the scent of commercial coffee and quickly categorize coffee samples [33]. GC-IMS was also used for the flavor compound analysis of sheep meat [34,35,36,37]. However, the use of GC-IMS to detect the freshness of goat meat has not been carefully studied.

In this study, GC-IMS combined with PCA and Euclidean distance analysis was used to distinguish the freshness of different samples of goat meat. The volatile basic nitrogen (TVB-N) contents and total viable counts (TVCs) of goat meat samples of different degrees of freshness were also detected and used for additional judgment. The results show that the multivariate, rapid, non-destructive evaluation model of goat meat freshness based on GC-IMS can effectively replace the traditional method used for goat meat freshness evaluation and that the method can be applied to an online inspection system. Our study shows that the freshness of goat meat can be detected to ensure the safety of meat supplies based on GC-IMS technology with the help of appropriate stoichiometric analysis methods.

## 2. Results

### 2.1. Experimental Results Obtained for TVB-N and TVC

**Figure 1:**
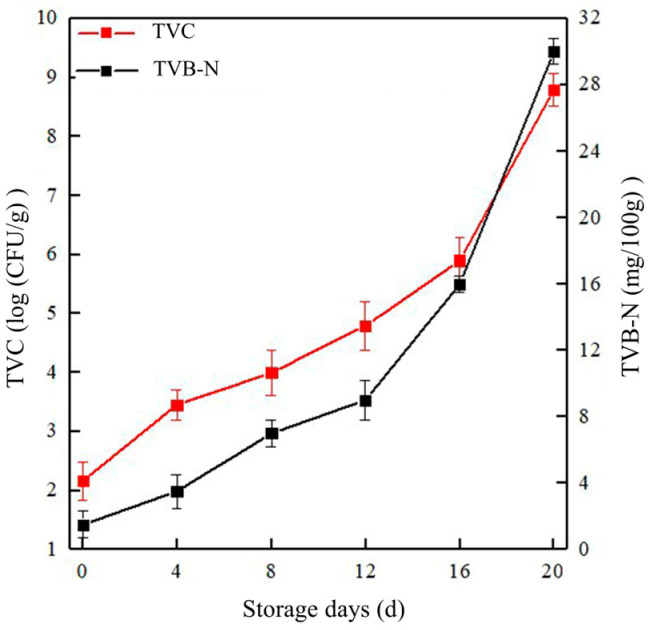
Changes in the TVC and TVB-N content of goat meat upon storage.

The TVB-N and TVC were tested by Chinese standards, and the results were shown in Figure 1. TVB-N refers to the organic nitrogen produced by protein breakdown and fatty acid abortion during the storage process of meat. [38]. As shown in Figure 1 (black line), the TVB-N values gradually increased upon extending the storage time. The TVB-N value of goat meat exhibited a significant growth rate after 16 d of storage (15.9 mg/100 g) and was above the limit of fresh meat (15 mg/100 g) in the current Chinese hygienic standard (GB 2707-2016) [39]. The TVC of the goat meat samples also showed an increasing trend upon storage (Figure 1, red line). According to the Chinese national food safety standard (GB/T 9961-2008), the upper limit of TVC in fresh and frozen goat meat is 5 log CFU/g. When refrigerated for 12 d, the TVC was close to this critical value.

### 2.2. GC-IMS Atlas Analysis

**Figure 2:**
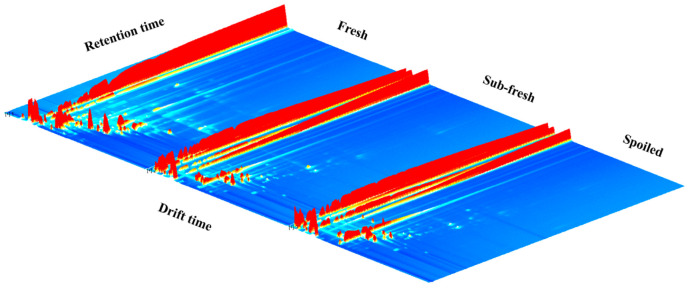
A comparison of the GC-IMS 3D spectra obtained for the fresh, sub-fresh, and spoiled meat samples.

According to the GC-IMS three-dimensional (3D) spectrogram (Figure 2), we found that the volatile organic compounds (VOCs) in fresh, sub-fresh, and spoiled meat samples exhibited significant differences in terms of ion peak signal intensity and quantity with time. All samples exhibited similar peak signal distributions, which indicated that similar volatile compounds were present after different storage times. However, with increased sample corruption levels, some VOCs showed different degrees of increase or decrease with increase in the storage time.

**Figure 3:**
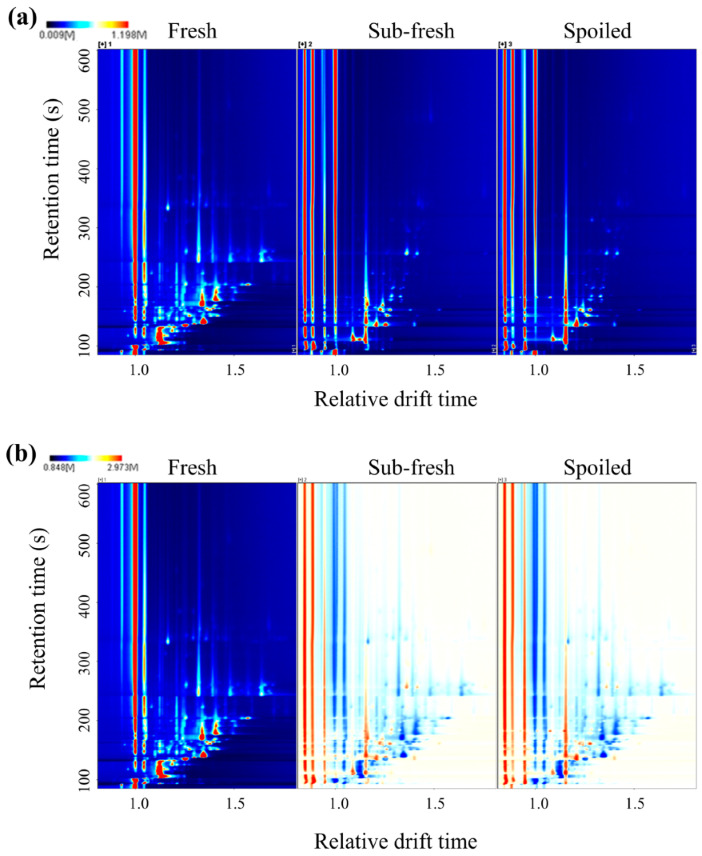
The two-dimensional top-view comparison of the VOCs for goat samples of different freshness: (**a**) ion mobility spectrogram; (**b**) results of comparison with the fresh sample were selected as the reference.

Figure 3 shows the GC–IMS two-dimensional (2D) spectra obtained for the goat meat samples of different freshness. The spectra show all of the volatile compounds in the samples. The ordinate is the retention time of the VOCs during the GC separation, the abscissa is the relative drift time of VOCs in the IMS separation when compared with the reaction ion peak, and the red vertical line at the abscissa 1.0 in Figure 3a is the reactive ion peak (RIP). Figure 3b shows the difference in samples observed by the difference comparison model [35,40]. The white and red shades of the spots indicate the degrees of accumulation and decomposition. White indicates lower intensity, and red indicates higher intensity. The intensity increases as the color deepens. Each point on either side of the RIP represents a VOC. Most signals appear in the hold time of 100–400 s and the drift time of 1.0–1.5 s.

The differences in goat meat samples were compared by applying a difference comparison model. The topographic map of fresh meat (left) was used as a reference, subtracted separately to produce the difference spectra of sub-fresh meat (middle) and spoiled meat (right). If the volatile components were consistent, the background after subtraction was white. Red indicates that the substance concentration was higher than the reference value, and blue indicates that the substance concentration was lower than the reference value; the darker the color, the greater the difference.

### 2.3. Analysis of the VOCs in Goat Meat

#### 2.3.1. Retention Index Distribution of VOCs in Goat Meat

**Figure 4:**
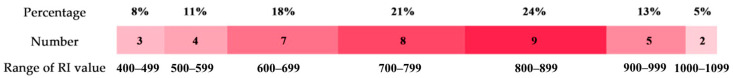
RI distribution of VOCs in the GC-IMS spectra of goat meat samples.

Table 1 and Figure 4 show that FlavourSpec^®^ can effectively capture VOCs with a low retention index (RI < 1000), accounting for 94.7%. When compared with GC-MS results, the incubation temperature, injection temperature, and column temperature results obtained via GC-IMS were lower [41,42]. Volatile and semi-volatile organic compounds (SVOCs) can be effectively captured and detected using IMS after pre-separation via GC during the upwelling process. Therefore, GC-IMS has obvious advantages in detecting VOCs in goat meat samples.

As shown in Table 1, all of the 38 identified VOCs could be divided into 9 groups, including 10 aldehydes, 10 ketones, 7 alcohols, 4 esters, 3 amines, 1 ether, 1 acid, 1 hydrocarbon, and 1 heterocyclic compound. The other 24 peaks were unidentifiable due to the imperfect library databases. Furthermore, higher proton affinities or higher concentrations will form dimers or multimers; six volatile compounds, including furfural, hexanal, 2-heptanone, 2-hexanone, 1-hexanol, and 1-pentanol, coexist as monomers (abbreviated as M) and dimers (abbreviated as D).

The main VOCs in fresh meat were (*E*)-2-heptene aldehyde, hexanal, furfural, amyl aldehyde, 3-methyl butyl aldehyde, 6-methyl-5-heptene-2-ketone, 2-ketone ketone, 2-heptanone, 2-hexanone, 2-pentanone, 3-hydroxy-2-butanone, 2-ethyl ketone, acetone and 1-hexanol, 1-amyl alcohol and isoamyl alcohol, 1-butyl alcohol, ethanol, 2-amyl furan, ethyl acetate, and methyl acetate. Dipropyl disulfide, ethyl 2-methylbutyrate, ethyl propionate, (*E*)-2-hexenal, octanal, and other substances were detected with an increase in the storage time (sub-fresh meat). Finally, higher concentrations of acetic acid, dimethylamine, trimethylamine, ammonia, and styrene were observed in spoiled goat meat. In particular, 1-Hexanol (flower, green, and resin odors) [43], 3-Hydroxy-2-butanone (sweet and sour odors) [44] and Ethyl Acetate (pineapple odor) [45] have a higher intensity in fresh meat than in un-fresh meat and can be used as identification indicators for fresh meat.

#### 2.3.2. Characteristic Distribution of VOCs in Goat Meat

**Figure 5:**
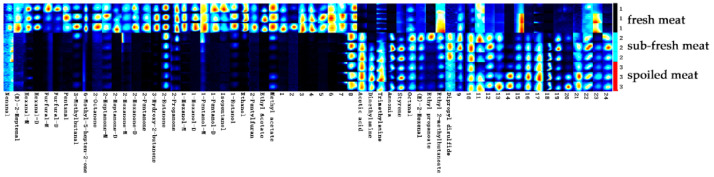
Fingerprints of volatile compounds in goat meat samples of different freshness.

To further compare the differences in the VOCs in goat meat samples of different freshness, the fingerprints of the volatile compounds in goat meat were established using the Gallery Plot plug-in, shown in Figure 5. Each row represents a sample, and each sample was measured three times in parallel, with all of the signal peaks of the VOCs contained in the sample at the same retention time and drift time.

From the ionic peak arrangement of the volatile substances shown in Figure 5, it can be seen that there are obvious similarities between the parallel sample groups and that the volatile substances in the goat meat samples with different degrees of freshness showed significant differences [46]. Taking fresh goat meat as a reference, there were significant differences in the types and concentrations of VOCs between sub-fresh meat and spoiled meat samples. There were similar changes in the types and concentrations of VOCs between the sub-fresh meat and spoiled meat samples, but there were also differences, which further indicated that GC-IMS could effectively distinguish the different freshness levels of the goat meat samples. On the whole, a variety of volatile substances found in fresh goat meat were significantly reduced with increasing storage time.

Combining Figure 5 and Table 1 (compound numbering is consistent with the fingerprints) shows that the VOC contents (including nonyl aldehyde, (*E*)-2-heptene aldehyde, hexanal, furfural, amyl aldehyde, 3-methyl butyl aldehyde, 6-methyl-5-heptene-2-ketone, 2-ketone ketone, 2-heptanone, 2-hexanone, 2-pentanone, 3-hydroxy-2-butanone, 2-ethyl ketone, acetone and 1-hexanol, 1-amyl alcohol and isoamyl alcohol, 1-butyl alcohol, ethanol, 2-amyl furan, ethyl acetate, methyl acetate, and other substances) were higher than those observed in the other samples.

The contents of dipropyl disulfide, ethyl 2-methyl butyrate, ethyl propionate, (*E*)-2-hexenal, octanal, and other substances in the sub-fresh meat samples were higher. The contents of acetic acid, dimethylamine, trimethylamine, ammonia, styrene, and other substances in the samples of sub-fresh meat and spoiled meat were higher.

### 2.4. Cluster Analysis of the Meat Samples

**Figure 6:**
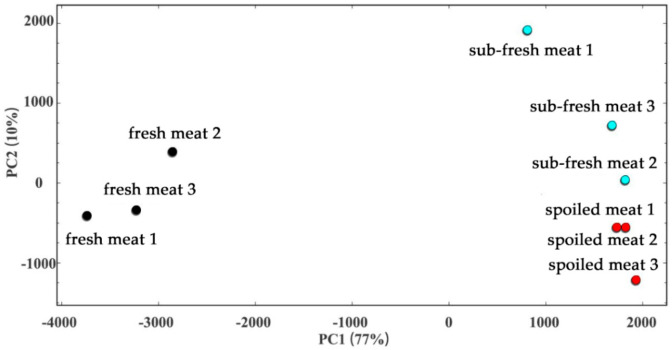
PCA analysis of VOCs found in goat meat samples of different freshness.

Principal component analysis (PCA) was used for dimensionality reduction of the 2D and 3D data obtained from GC-IMS, in order to visually analyze the characteristics of frozen goat meat samples with different storage times. In general, when the cumulative contribution rate reaches 60%, the PCA model can be used as the separation model [47,48,49]. Figure 6 shows that the sum of the contribution rates of principal components 1 and 2 was >80%, and the comprehensive variables obtained after reduction of the dimensionality can express most of the information of the original variables in a two-dimensional space. When the cumulative variance contribution rates were 77% and 10%, the two major components of mutton at different freshness levels were clearly separated. The difference between each sample in the group is relatively concentrated in a certain range, and other groups of data clusters show clear spacing.

As shown in the PCA distribution map, it can be clearly seen that, in a completely independent space, goat meat samples of different freshness can be well distinguished in the visualization. Fresh meat can be distinguished according to negative score values for PC1 and negative score values for PC2 (except the fresh meat 2 sample). Sub-fresh meat can be distinguished by positive score values for PC1 and positive score values for PC2. Spoiled meat can be distinguished by positive score values for PC1 and negative score values for PC2. These results also suggest that storage time results for the same sample have good repeatability and that the specificities of samples with different storage times are more obvious.

**Figure 7:**
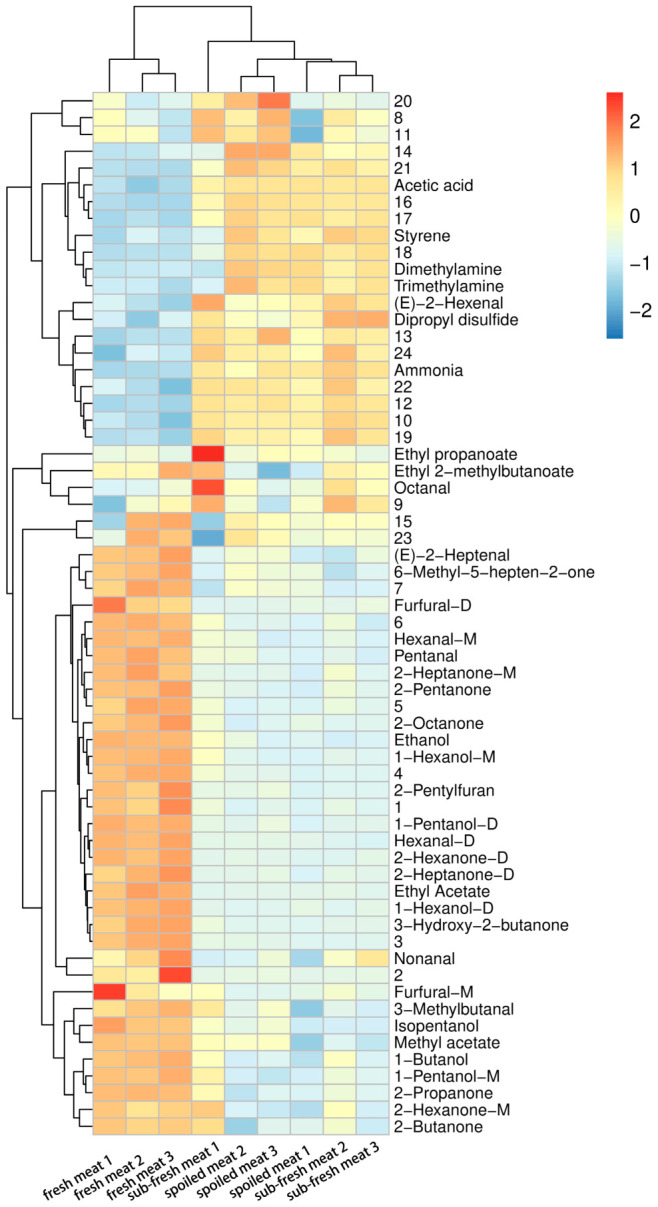
Heat map clustering of volatile substances produced during storage of the goat meat samples.

The R program was used to further analyze the VOCs in goat meat samples under different storage conditions (Figure 7). The horizontal clustering results of the clustering heat map represent the relative content relationships between samples of different freshness degrees. The cluster analysis results showed that the samples of sub-fresh meat and spoiled meat could be grouped into one class. The changes in acetic acid, styrene, dimethylamine, trimethylamine, and other substances showed regularity in un-fresh meat. Acid compounds are more easily detected in long-term stored meat, the production of which can be initiated by enzymes or microorganisms existing in the goat meat [50]. When compounds containing nitrogen, including Dimethylamine, trimethylamine, and ammonia, exceed a particular threshold in goat meat, an offensive stench is produced. The detection of volatile organic nitrogen compounds at high concentrations indicates the massive degradation of proteins and amines [51]. This tendency of changes in TVB-N levels was also noticed. Unsurprisingly, as the meat spoiled further, the relative concentrations of nonanal, 3-methyl butanal, 1-butanol, and other chemicals declined. The changes in typical flavor substances make it easy for us to distinguish between fresh and un-fresh goat meat by GC-IMS.

**Figure 8:**
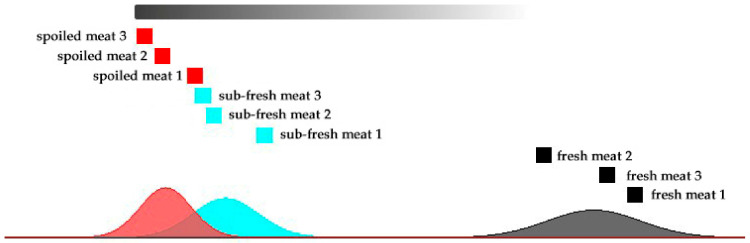
Nearest-neighbor Euclidean distance diagram for goat meat samples of different freshness.

The data processed using PCA were applied to cluster analysis, and goat meat samples with known storage times were used as test samples to test the classification effect. Due to the small number of samples in each storage period, the nearest-neighbor algorithm was used for the analysis. Figure 8 shows that goat meat samples at the same stage had high similarity and close sample distances. Therefore, the analysis using the nearest-neighbor algorithm was suitable for the discrimination of goat meat freshness.

## 3. Discussion

Based on GC-IMS detection, the levels of volatile components in goat meat samples of different freshness changed significantly. According to our results, there were 62 VOCs, but, due to the imperfect database, only 38 volatile substances had qualitative results; the remaining 24 VOCs need further study. There were 32 monomers in 38 volatile substances, including 5 alcohols, 8 ketones, 8 aldehydes, 4 esters, and 7 others. Fresh meat contains more alcohol and aldehyde VOCs. Alcohols were common flavor substances found in various meats, such as fish [52,53], chicken [54], pork [55], duck [56], and so on. Alcohols mainly originate from the oxidation of unsaturated fats, and the threshold value of unsaturated alcohols is low, which has a great influence on flavor; 1-Amyl alcohol, for example, has a green odor [57]. Aldehydes mainly originate from lipid oxidation and have lower thresholds, which plays a crucial role in the flavor of meat [34]. Hexanal and pentaldehyde had berry, nut, and fruit fragrances; furfural had a sweet popcorn and wood odor; while nonanal and (*E*)-2-heptenal had a fatty fragrance [24,25,57]. Ketones are derived from the Maillard reaction and fat degradation [26]. 2-Heptanone, 2-pentanone, and 2-butanone have the aromas of banana, wood, and sweet coconut fruit, respectively, and aldehydes give lamb a pleasant aroma.

Upon extending the storage period, alcohols and aldehydes rich in fruit flavors and fragrances were no longer detected in the sub-fresh meat, and flavor began to change dramatically. Only ethyl propionate, octyl aldehyde, and other substances with good flavors of fat and fruit were detected. Ethyl acetate, which has a fruity aroma, underwent a significant reduction during storage, which is common in fresh meat [58].

In addition, the rancid odors of dimethylamine, trimethylamine, ammonia, and other substances were detected in large quantities in the spoiled meat samples, indicating that the meat was seriously corrupted. Figure 6 shows that dimethylamine, trimethylamine, and other volatile components exhibited obvious increasing trends with an increase in storage time. The dimethylamine, trimethylamine, acetic acid, and other characteristic flavor peak regions of sub-fresh meat and spoiled meat were obviously different from those of fresh meat and can be used as characteristic fingerprint regions to identify spoiled meat. Therefore, the storage time of goat meat can be judged by the increase and decrease in VOCs and the relative change in the concentration of these substances.

The physiological and biochemical metabolism of fresh goat meat during storage will make the quality of fresh goat meat change rapidly. When combining the total number of bacteria and detection results for volatile base nitrogen (TVB-N), the quality of goat meat reached the sub-fresh stage when it was stored for 13 d. After 17 d of preservation, the goat meat seriously deteriorated and became spoiled. Based on GC-IMS fingerprint analysis, dimethylamine, trimethylamine, acetic acid, ammonia, styrene, and other characteristic volatile components can be used to identify the freshness and chilled storage time of goat meat.

## 4. Materials and Methods

### 4.1. Materials

Fresh goat meat samples (the cleaned hind leg meat of the goat was cut into small pieces 1 × 1 × 1 cm in size) were purchased from RT-Mart in Bengbu, placed in an insulated box at low temperature, and brought back to the laboratory. Plate Count Agar (PCA) was purchased from Solarbio (Beijing, China). All other chemicals and solvents were obtained from were purchased from Adamas-beta Reagent Co., Ltd. (Shanghai, China).

### 4.2. Sample Processing

The same batch of goat meat was divided into the control group and experimental group. Seven batches of goat meat from the same source were prepared, marked for subsequent detection, and sealed at 4 °C for storage.

### 4.3. Determination of TVB-N and TVCs

The method used for the determination of TVB-N in chilled goat meat was performed according to the Chinese standard GB 5009.228-2016 [59]. The determination of the TVB-N of the goat meat samples was performed on an Automatic Kjeldahl nitrogen analyzer (K9860, Hanon, Jinan, China). The results were expressed as TVB-N (mg/100 g).

The TVC of the goat meat samples was determined according to a method described in the literature [60]. A sample of 5 g of goat meat was sterilely weighed and homogenized with 45 mL of bacteria-free 0.85% NaCl solution for 1 min. From this dilution, other decimal dilutions were prepared using 0.85% NaCl solution. Then, 0.1 mL of the diluted samples was spread onto plate count agar for the enumeration of the TVCs. The inoculated plates were then incubated for 72 h at 30 °C for TVC analysis. All counts were carried out in duplicate, and the results were expressed as log CFU/g.

### 4.4. The experimental Method of GC-IMS

#### 4.4.1. Instrumentation

The analyses of goat meat samples were completed on an IMS instrument (FlavourSpec^®^ Gesellschaft für Analytische Sensorsysteme mbH, Dortmund, Germany) equipped with an autosampler unit. The specifications of the chromatographic column were as follows: MXT-5; 15 m × 0.53 mm ID; film thickness, 1 μm.

#### 4.4.2. GC-IMS Conditions

Headspace incubation temperature, 60 °C; incubation time, 15 min; incubation speed, 500 rpm; injection volume, 100 μL; injection needle temperature, 65 °C; non-shunt mode; cleaning time, 0.50 min. The carrier gas was high-purity N_2_ (≥99.999%). The chromatographic column temperature was 60 °C, and the chromatographic running time was 20 min. The flow rate gradient of carrier gas was set to 2.00 mL/min, kept for 2 min, and linearly increased to 20.00 mL/min over 6 min. It was linearly increased to 100.00 mL/min over 2 min and kept for 5 min.

#### 4.4.3. Detection Method

Fresh meat samples, secondary fresh meat samples kept fresh for 12 days, and stale meat samples kept fresh for 17 days were selected. After crushing, 2 g meat samples were weighed and placed in a 20 mL headspace bottle for incubation. The headspace components in the bottle were extracted with a heated injection needle, and the volatile components were analyzed using a FlavourSpec^®^ flavor analyzer.

#### 4.4.4. Data Analysis

Commercial VOCal software (0.2.9, G.A.S. Gesellschaft für analytische Sensorsysteme mbH, Dortmund, Germany) was used for the qualitative and quantitative analysis of the spectrograms and data. The NIST database and IMS database built-in software systems were used for qualitative analysis of the substances. Each of the dots represents a VOC. The reporter plug-in was used to directly compare the spectral differences between the samples (3D spectral, 2D top view, and differential spectral). The topographic plot of the fresh meat sample was selected as a reference, and the topographic plots of the other samples were deducted from the reference. The white and red shades of the spots indicate the degrees of accumulation and decomposition. The ordinate is the retention time of VOCs in the GC separation, the abscissa is the relative drift time of the VOCs in the IMS separation compared to the reaction ion peak, and the red vertical line at abscissa 1.0 is the RIP (normalized). The VOCs of the different samples were compared visually and quantitatively using Gallery Plot: Fingerprint comparison. Dynamic PCA was performed using the Dynamic PCA plug-in, which was used for cluster analysis of the samples and rapid determination of the unknown samples. Nearest-neighbor fingerprint analysis was used to discover the nearest neighbors by calculating the Euclidean distances between two samples and retrieving the minimum distance, which was used to evaluate the strength of compounds in the region and make a rapid comparison of the samples.

## 5. Conclusions

In order to develop a method for rapidly detecting goat meat freshness, this study used GC-IMS technology to determine the volatile components in goat meat at different periods of storage combined with physical and chemical experiments to assess TVB-N contents and total numbers of bacteria to determine fingerprints for fresh meat, sub-fresh meat, and spoiled meat. The results of our fingerprint analysis showed that the dimethylamine, trimethylamine, acetic acid, and other characteristic volatile component peak regions of sub-fresh meat and spoiled meat were significantly different from those of fresh meat. These can be used as characteristic fingerprint regions for identifying spoiled meat. Dynamic principal component analysis and nearest-neighbor Euclidean distances can be used to analyze the differences between different grades of meat, which can be used to quickly determine the freshness of goat meat.

## Figures and Tables

**Table 1:** Specific information on the volatile compounds in goat meat samples of different freshness.

Compounds	MW	RI	Rt (s)	Dt (RIPrel)	The Peak Intensity		
					Fresh Meat	Sub-Fresh Meat	Spoiled Meat
Nonanal	142.2	1109.4	507.00	1.47224	134.67 ± 16.47	112.6 ± 14.37	100.36 ± 7.2
6-Methyl-5-hepten-2-one	126.2	982	332.67	1.16872	819.63 ± 117.43	199.81 ± 26.1	292 ± 31.68
2-Pentylfuran	138.2	994.4	343.20	1.26022	152.99 ± 57.26	20.31 ± 2.71	21.56 ± 4.07
2-Octanone	128.2	988.9	338.52	1.35933	201.15 ± 53.1	30.03 ± 8.61	25.28 ± 4.49
2-Heptanone-M	114.2	894.4	258.57	1.25869	277.81 ± 43.47	80.54 ± 15.88	67.6 ± 5.84
2-Heptanone-D	114.2	894	258.18	1.63533	651.06 ± 315.24	35.53 ± 1.95	35.14 ± 7.79
1-Hexanol-M	102.2	872.6	246.48	1.32121	1367.78 ± 204.33	138.8 ± 68.68	88.76 ± 11.05
1-Hexanol-D	102.2	869.6	244.92	1.63838	1027.97 ± 298.11	36.27 ± 5.67	35.33 ± 6.1
(*E*)-2-Heptenal	112.2	954.4	309.27	1.26022	87.47 ± 17.56	17.1 ± 4.38	20.72 ± 5.89
Furfural-M	96.1	829.1	223.86	1.08333	228.79 ± 201.4	77.46 ± 14.29	59.18 ± 2.91
Furfural-D	96.1	828.3	223.47	1.34408	379.64 ± 260.16	38.96 ± 4.74	38.45 ± 4.11
Hexanal-M	100.2	791.3	204.23	1.25743	769.3 ± 78.18	108.25 ± 30.82	91.89 ± 28.01
Hexanal-D	100.2	791.3	204.23	1.56718	951.23 ± 239.23	21.78 ± 4.46	24.85 ± 1.3
2-Hexanone-M	100.2	781.7	199.51	1.1807	67.42 ± 8.89	40.13 ± 28.33	15.4 ± 2.08
2-Hexanone-D	100.2	781	199.27	1.49755	370.76 ± 77.81	19.05 ± 2.6	19.47 ± 2.54
1-Pentanol-M	88.1	763.4	192.06	1.24606	506.38 ± 62.43	141.63 ± 74.77	69.22 ± 5.47
1-Pentanol-D	88.1	759.7	190.57	1.51176	361.02 ± 52.76	19.02 ± 2.61	17.43 ± 4.73
Isopentanol	88.1	728	177.66	1.49613	332 ± 117.62	27.71 ± 18	27.62 ± 12.22
3-Hydroxy-2-butanone	88.1	709.3	170.04	1.33844	3349.22 ± 1203.65	140.62 ± 26.97	110.76 ± 6.14
Pentanal	86.1	693.8	163.75	1.42336	97.36 ± 27.32	4.41 ± 2.02	4.22 ± 1.72
2-Pentanone	86.1	693	163.40	1.36302	495.65 ± 176.86	19.57 ± 5.45	12.47 ± 2.47
1-Butanol	74.1	685.4	160.79	1.37978	411.35 ± 57.75	76.87 ± 32.02	34.27 ± 7.71
3-Methylbutanal	86.1	648.4	151.01	1.41777	320.47 ± 105.92	77.03 ± 80.15	36.6 ± 25.94
Ethyl Acetate	88.1	608.7	140.53	1.34403	4009.34 ± 1618.55	63.88 ± 7.93	71.83 ± 0.62
2-Butanone	72.1	588.2	135.12	1.25353	3265.26 ± 130.9	2135.66 ± 826.26	1461.21 ± 234.95
Methyl acetate	74.1	551.2	125.34	1.18872	661.91 ± 39.34	89.72 ± 70.73	96.34 ± 62.91
2-Propanone	58.1	500.9	112.06	1.12057	6216.66 ± 86.68	1936.03 ± 675.44	1212.88 ± 178.14
Ethanol	46.1	469.8	103.86	1.12839	3654.02 ± 57.73	263.72 ± 179.61	201.85 ± 59.64
Acetic acid	60.1	612.7	141.58	1.15967	224.97 ± 48.26	1711.15 ± 333.28	2011.53 ± 64.07
Ethyl propanoate	102.1	689.1	161.83	1.45576	7.21 ± 0.8	32.41 ± 42.79	9.53 ± 1.53
Dimethylamine	45.1	446	97.57	0.95409	163.36 ± 14.1	1005.22 ± 844.72	2576.22 ± 418.3
Trimethylamine	59.1	520.7	117.30	1.15855	1330.47 ± 138.29	2587.24 ± 1048.38	4036.3 ± 580.56
Ammonia	17	447.4	97.92	0.89375	392 ± 18.72	4593.52 ± 1263.04	3087.01 ± 1186.73
Styrene	104.2	891.1	256.14	1.41659	125.45 ± 15.61	262.75 ± 103.59	283.26 ± 60.9
Octanal	128.2	991.5	340.73	1.42632	120.71 ± 5.98	168.41 ± 35.68	126.78 ± 7.55
(*E*)-2-Hexenal	98.1	824.7	221.60	1.19013	24.45 ± 5.18	118.97 ± 29.93	57.1 ± 9.06
Ethyl 2-methylbutanoate	130.2	826.9	222.76	1.24848	103.69 ± 23.04	103.76 ± 19.55	62.29 ± 9.9
Dipropyl disulfide	150.3	1094	484.99	1.48917	47.67 ± 7.68	125.7 ± 20.48	73.99 ± 7.63

Note: RI = retention index, Rt = retention time, and Dt = migration time.

## Data Availability

The data presented in this study are available in article.

## Sample Availability

Samples of the goat meat are available from the authors.
